# Effective breastfeeding techniques and associated factors among lactating women: a community-based study, north east Ethiopia

**DOI:** 10.3389/fpubh.2024.1337822

**Published:** 2024-03-21

**Authors:** Esuyawkal Mislu, Henok Kumsa, Mulugeta Wodaje Arage, Anguach Shitie, Abebe Adimasu

**Affiliations:** ^1^Department of Midwifery, College of Health Science, Woldia University, Woldia, Ethiopia; ^2^Department of Midwifery, College of Medicine and Health Science, Wollo University, Woldia, Ethiopia

**Keywords:** effective breastfeeding techniques, good attachment, good positioning, good suckling, breastfeeding

## Abstract

**Background:**

Effective breastfeeding techniques, which include proper attachment, positioning, and suckling, offer a range of benefits for both the mother and the infant. These techniques ensure efficient milk transfer, reduce the risk of infections, support optimal infant weight gain, enhance maternal comfort, and foster a strong emotional bond. This study aimed to identify the magnitude and factors associated with effective breastfeeding techniques among lactating women in the Legambo district of South Wollo, Ethiopia, in 2022.

**Methods:**

A community-based cross-sectional study was conducted from September to November 2022. Samples were selected using a multi-stage sampling method from 18 wards (kebele). Data were collected using an interviewer-administered structured questionnaire and an observational checklist. The collected data were entered into Epi-Data and then exported to SPSS version 25.0 for analysis. Descriptive statistics and bivariate and multivariable logistic regression analyses were performed to identify the magnitude and associated factors. Variables with a *p*-value less than 0.05 on multivariable analysis were considered independent factors associated with the outcome variable.

**Results:**

Six hundred and ten lactating women were included for observation and interviewed, resulting in a 96.2% response rate. The magnitude of effective breastfeeding technique practice was found to be 25.9% (95% CI: 22.47–29.57%). Factors associated with effective breastfeeding technique practice included being a working woman (AOR = 1.70; 95%CI: 1.07–2.72), age between 26 and 30 years (AOR = 0.37; 95%CI: 0.16–0.84), urban residence (AOR = 1.59; 95%CI: 1.06–2.39), initiating breastfeeding 1 to 2 h after birth (AOR = 0.27; 95%CI: 0.16–0.43), and initiating breastfeeding after 2 h of birth (AOR = 0.34; 95%CI: 0.17–0.67). Additionally, not receiving breastfeeding education (AOR = 0.46; 95%CI: 0.30–0.72) and experiencing current breast problems (AOR = 0.28; 95%CI: 0.28–0.75) were also found to have a significant association with effective breastfeeding technique practice.

**Conclusion:**

Only one in four women demonstrated effective breastfeeding techniques, indicating that their practice was below the WHO’s recommendations. Therefore, it is crucial to consider the identified variables to improve the practice of effective breastfeeding techniques.

## Introduction

Breastfeeding is the practice of feeding a child directly from the breast or through expressed milk by hand or with a pump (1, 2). It is a natural and essential practice that provides optimal nutrition and promotes the overall health and development of infants (1–3). The World Health Organization (WHO) and the Ethiopian Federal Ministry of Health recommend initiating breastfeeding within the first hour of a baby’s life and exclusively breastfeeding for the first 6 months. After 6 months, breastfeeding should be continued along with appropriate complementary feeding for up to 2 years and beyond (1, 2). Implementing these recommendations has the potential to prevent approximately 820,000 deaths of children under the age of 5 annually in low- and middle-income countries, where the burden of under-five mortality is high (4).

Breastfeeding has numerous short-term and long-term benefits for the mother and the child. It decreases the risk of infection, sudden infant death syndrome, asthma, food allergies, and diabetes for the baby while also improving cognitive development and decreasing the risk of obesity in adulthood (4–6). For the mother, breastfeeding offers benefits such as a decrease in postpartum hemorrhage, depression, and facilitation of involution. In the end, it also reduces the risk of breast cancer, cardiovascular disease, diabetes, metabolic syndrome, and rheumatoid arthritis (6–8).

Effective breastfeeding technique is a process of breastfeeding that occurs when the baby and mother show readiness for it, and the baby is appropriately positioned, has good attachment, and has good suckling (9, 10). Effective breastfeeding techniques are essential for women to feed their babies without pain, in a simple, safe, and time-saving manner (9). Proper attachment, positioning, and suckling during breastfeeding offer a range of benefits for both the mother and the infant. These components ensure efficient milk transfer, prevent nipple soreness and discomfort, reduce the risk of infections, support optimal infant weight gain, and enhance maternal comfort (10, 11).

Furthermore, practicing effective breastfeeding techniques fosters a strong emotional bond between the mother and the baby (9). Physical closeness, eye contact, and skin-to-skin contact during breastfeeding stimulate the release of oxytocin, a hormone that promotes feelings of love, nurturing, and attachment. This bonding experience contributes to the emotional wellbeing of both the mother and the infant (12). Emphasizing and supporting this aspect of breastfeeding is crucial to enhance successful breastfeeding outcomes and improve the overall health and wellbeing of both the mother and infant (10–12).

Promoting effective breastfeeding practices is of utmost importance in Ethiopia, where infant mortality rates remain a significant concern. However, there is a notable disparity in the prevalence and factors associated with effective breastfeeding techniques among different populations. Furthermore, most previous studies have been conducted in institutional settings. Therefore, this study aims to bridge these gaps by investigating the magnitude and factors associated with effective breastfeeding among lactating women in the Legambo district of South Wollo, Ethiopia, in 2022. The findings from this study will provide valuable insights to develop targeted interventions aimed at improving breastfeeding outcomes and enhancing the overall health and wellbeing of both mothers and infants, reducing healthcare costs, and fostering a sustainable environment in the region.

## Materials and methods

### Study setting

A community-based cross-sectional study was conducted in Legambo District, South Wollo Zone, from September to November 2022. The district is situated 450 km away from Bahir Dar, the capital city of the Amhara region, and 601 km from Addis Ababa, the capital city of Ethiopia. Legambo District comprises 41 wards, of which 38 are rural wards (kebeles) and three are urban wards. The district is equipped with 1 general hospital, 9 health centers, and 39 health posts (13).

### Study population

The study included all lactating women who had given birth in the past 6 weeks and were living in selected wards of the district.

### Eligibility criteria

All postnatal women who were breastfeeding during the study period were included. However, mothers of infants with serious illnesses that affect breastfeeding, such as inability to suck, lethargy, and unconsciousness, were excluded.

### Sample size determination

The sample size was calculated using the single population proportion formula, assuming a 95% confidence interval, a 5% margin of error, and an estimated magnitude of effective breastfeeding of 48% based on a previous study in Gondar town (14). The margin of error was set at 1.5.

The formula used was: Sample size = [(Z1-α/2)2*P(1-P)]/d2.

where:

- (Z1-α/2) is the standard normal variation.
- P is the proportion in the population (48%).
- d is the margin of error (0.05).

The calculated sample size without considering design effect and non-respondents was 384. Taking into account a design effect of 1.5 and a 10% non-response rate, the final sample size was determined to be 634 postnatal mothers.

### Sampling procedure

A multi-stage sampling method was employed. Initially, 18 wards (16 rural and 2 urban) were selected from the total using the lottery method. The target sample size was then proportionally allocated to each of the selected wards. Finally, study participants were recruited using a simple random sampling method, with the registration book at health posts serving as the sampling frame.

### Operational definition

- *Effective breastfeeding technique*: The achievement of at least two criteria from positioning, three criteria from attachment, and two criteria from suckling while breastfeeding their infant (15).
- *Breast problems*: A woman who had one or more of the following six conditions: inverted nipple, flat nipples, cracked nipples, engorgement, mastitis, and breast abscess.
- *Poor, average, and good positioning*: Women who fulfill none or only one, any two, and all four/three out of four criteria were considered as poor, average, and good positioning, respectively (15). The criteria were as follows: the baby’s head and body are in line; the baby is held close to the body; the baby’s whole body is supported with the arm along their back; and the baby approaches breast nose to the nipple so that it comes to the breast from underneath the nipple (15).
- *Poor, average, and good attachment*: Women who fulfilled none or only one criterion, any two criteria, and all four/three criteria out of four criteria for infant attachment were considered as poor, average, and good attachment, respectively (15).
- *Poor suckling*: Women who do not achieve any or only one of the three criteria (slow and regular suckling, deep suckling, and sometimes pausing) (15).
- *Deep suckling*: A suckling demonstrated by visible or audible swallowing after every one or two sucks (14).
- *Good knowledge about the benefits of breastfeeding*: Women who correctly answered seven or more out of 13 questions were considered to have good knowledge about the benefits of breastfeeding (16).

### Data collection tools and procedure

Data were collected using an interviewer-administered structured questionnaire and an observational checklist. The questionnaire included socio-demographic, obstetric, breastfeeding, and breast problem-related questions. The observational checklist was used to assess the woman’s and baby’s position, attachment, and suckling during breastfeeding, following the WHO’s breastfeeding checklist (14, 17). The data collection tools were developed after reviewing relevant literature to address important variables related to the main objective (12, 16, 18–22). Ten trained female bachelor (BSc.) degree midwives and two supervisors were employed as data collectors. They observed the breastfeeding practice of each mother for 4 min and recorded it on the observation checklist form. Written informed consent was obtained from all participants before the interview and observation, and confidentiality was assured throughout the study process.

### Data quality control

To ensure data quality, both the data collectors and supervisors received 2 days of training before data collection. Language experts translated the English version of the questionnaire into the local language (Amharic). Additionally, a pre-test was conducted on 5% (32 mothers) of the sample size before the actual data collection. The tool was modified and corrected based on the pre-test results. Each questionnaire was checked for completeness before data entry, and the data were then edited, coded, and entered into Epi Data version 4.6.1. Double entry was performed, and entry errors were checked and corrected accordingly.

### Data processing and analysis

The data were analyzed using SPSS version 25.0. Descriptive statistics such as frequency, percentage, mean, and standard deviation were calculated and presented in tables, figures, and text. Bivariate and multivariable logistic regression analyses were conducted to identify factors associated with effective breastfeeding. Variables with *p*-values less than 0.25 in the bivariate analysis were included in the multivariable logistic regression to examine the relative effect of confounding variables and variable interactions. Variables with *p*-values less than 0.05 in the multivariable analysis were considered independent factors associated with the outcome variable. The omnibus test of model coefficients and the Hosmer and Lemeshow goodness-of-fit test were used to assess model fitness, with *p*-values of 0.000 and 0.851, respectively. Variance inflation factor (VIF) and tolerance tests were also conducted to check for multicollinearity among the explanatory variables, with the highest VIF at 1.18 and the lowest tolerance test at 86.7%.

## Results

### Socio-demographic characteristics of study participants

Six hundred and ten lactating women were observed and interviewed for effective breastfeeding, with a response rate of 96.2%. The participants’ mean age (**±**SD) was 25.69 (**±**6.014) years. The majority of respondents, 471 (77.2%), were housewives, 327 (53.6%) were rural dwellers, and 545 (89.3%) were married. Only 115 (18.8%) had a secondary or higher degree of education (Table 1).

**Table 1:** Socio-demographic characteristics of study participants in Legambo District, North-East Ethiopia, 2022 (*n* = 610).

Variables	Frequency	%
*Age of the mothers*		
<20	67	11.0
20–25	278	45.6
26–30	162	26.6
˃30	103	16.9
*Residence*		
Rural	327	53.6
Urban	283	46.4
*Level of education*		
No formal education	266	43.6
Primary education	229	37.7
Secondary education	80	13.1
More than secondary	35	5.7
*Marital status*		
Married	545	89.3
Not married	65	10.7
*Occupation*		
Housewife	471	77.2
Working	139	22.8

### Obstetrics characteristics of the respondents

Three-hundred and eighty (62.3%) of the total participants were multipara, and 56 (9.2%) gave birth at home. Seventy-five percent (456%) of the study participants received prenatal care (ANC). In terms of mode of delivery, 517 (84.8%) were spontaneous vaginal deliveries. Infants were born at term in 534 (87.5%) of the cases, and 554 (90.8%) of postpartum women had no history of breast problems (Table 2).

**Table 2:** Obstetrics characteristics of the participants for the study conducted in Legambo District, North East Ethiopia, 2022 (*n* = 610).

Variables	Frequency	Percent (%)
*Parity*		
Primipara	230	37.7
Multipara	380	62.3
*Place of birth*		
Home	56	9.2
Health institution	554	90.8
*Mode of delivery*		
Vaginal delivery	517	84.8
Cesarean delivery	93	15.2
*GA in complete weeks*		
Preterm	104	17.0
Term	506	83.0
*Previous history of breast problems*		
Yes	56	9.2
No	554	90.8
*ANC follow-up*		
Yes	456	74.8
No	154	25.2
*PNC follow-up*		
Yes	500	81.97
No	110	18.03
GA-Gestational age		

### Breastfeeding characteristics of the study participants

Merely 74 (12.1%) of the total participants began breastfeeding within an hour after birth, whereas 236 (61.3%) of them got health education on the topic. Approximately 38.2% of the total participants were postpartum nursing mothers who mixed-fed their kids at the time of the interview with other foods and liquids in addition to breast milk (Table 3).

**Table 3:** Breastfeeding characteristics of the participants for the study conducted in Legambo District, North East Ethiopia, 2022 (*n* = 610).

Variables	Frequency	Percent (%)
*Received BF education*		
No	236	38.7
Yes	374	61.3
*Time of breastfeeding initiation*		
Within 1 h	174	28.5
2–3 h	384	63
First day (4–24 h)	49	8
At or After 24 h	3	0.5
*Type of feeding*		
Exclusive breastfeeding	377	61.8
Mixed feeding	233	38.2
*Previous breastfeeding experience*		
Yes	295	48.4
No	315	51.6
*Mother knowledge on benefits of BF*		
Good knowledge	346	56.7
Poor knowledge	264	43.3
*Source of information on breastfeeding*		
Friends and family	341	55.9
Healthcare providers	160	26.2
Audiovisual*	109	17.9
*Breast problem*		
Yes	316	54.3
No	294	45.7

Audiovisual*: Radio, TV, Newspaper, and Internet.

### Magnitude of effective breastfeeding

In this study, 358 (58.7%), 337 (55.2%), and 416 (68.2%) women demonstrated good positioning, attachment, and suckling, respectively. The practice of effective breastfeeding techniques was identified among 158 women, which accounts for a magnitude of 25.9% with a 95% confidence interval ranging from 22.47 to 29.57%. The remaining 452 (74.1%) women exhibited ineffective breastfeeding practices.

### Factors associated with effective breastfeeding

Bi-variable and multivariable logistic regressions were performed. In the bi-variable analysis, 14 variables (occupation, age of mothers, residence, marital status, place of birth, parity, gestational age, mode of delivery, previous breast problem history, receiving BF education, breastfeeding experience, type of feeding, current breast problem, and BF initiation time) had *p*-values less than 0.25. These 14 variables were adjusted to each other on the multivariable logistic regression to identify the AOR with their 95% confidence interval. In the multivariable logistic regression analysis, age, residence, and occupation of the mothers, breastfeeding initiation time, receiving BF education, and current breast problem retained their association with effective breastfeeding at a *p*-value less than 0.05 (Table 4).

**Table 4:** Bi-variable and multivariable analysis of factors associated with effective breastfeeding techniques among postnatal lactating mothers in Legambo District, North East Ethiopia, 2022 (*n* = 610).

Variables		Effective breastfeeding		COR (95%CI)	AOR (95%CI)	*p*-value for AOR
		No	Yes			
Occupation	Housewife	360 (76.4%)	111 (23.6%)	Ref.	Ref.	
	Working	92 (66.2%)	47 (33.8%)	1.65 (1.09–2.49)	1.70 (1.07–2.72)*	**0.024**
Age of mothers	≤ 20	48 (70.6%)	20 (29.4%)	0.94 (0.42–2.10)	0.60 (0.23–1.50)	0.278
	20–25	173 (71.2%)	70 (28.8%)	0.91 (0.47–1.78)	0.76 (0.36–1.61)	0.477
	26–30	140 (80.5%)	34 (19.5%)	0.55 (0.27–1.12)	0.37 (0.16–0.84)*	**0.018**
	30–35	57 (75%)	19 (25%)	0.75 (0.34–1.68)	0.69 (0.28–1.68)	0.415
	≥35	34 (69.4%)	15 (30.6%)	Ref.	Ref.	
Residence	Rural	255 (78%)	72 (22%)	Ref.	Ref.	
	Urban	197 (69.6%)	86 (30.4%)	1.54 (1.07–2.22)	1.59 (1.06–2.39)*	**0.024**
Marital status	Married	399 (73.2%)	146 (26.8%)	Ref.	Ref.	
	Not married	53 (81.5%)	12 (18.5%)	0.61 (0.32–1.19)	0.50 (0.24–1.05)	0.068
Place of birth	Home	47 (87.0%)	7 (13.0%)	0.39 (0.17–0.90)	0.50 (0.20–1.27)	0.148
	HI^a^	405 (72.8%)	151 (27.2%)	Ref.	Ref.	
Parity	Primi	180 (78.6%)	49 (21.4%)	Ref.	Ref.	
	Multi	272 (71.4%)	109 (28.6%)	1.47 (1.00–2.16)	0.78 (0.47–1.28)	0.327
Gestational age	Preterm	44 (84.6%)	8 (15.4%)	0.49 (0.22, 1.07)	1.12 (0.45–2.74)	0.800
	Term	408 (73.1%)	150 (26.9%)	Ref.	Ref.	
Delivery mode	Vaginal	388 (75%)	129 (25%)	Ref.	Ref.	
	CS	64 (68.8%)	29 (31.2%)	1.36 (0.84–2.20)	1.58 (0.89–2.72)	0.115
Hx^b^ of breast problem	Yes	33 (58.9%)	23 (41.1%)	2.16 (1.22–3.81)	0.83 (0.42–1.63)	0.601
	No	419 (75.6%)	135 (24.4%)	Ref.	Ref.	
Breastfeeding initiation time	<=1 h	98 (57%)	74 (43%)	Ref.	Ref.	
	1–2 h	295 (81.5%)	67 (18.5%)	0.30 (0.20–0.45)	0.27 (0.16–0.43)***	**0.000**
	>2 h	59 (77.6)	17 (22.4%)	0.38 (0.20–0.70)	0.34 (0.17–0.67)**	**0.002**
Receiving BF education	No	190 (80.9%)	45 (19.1%)	0.54 (0.37–0.81)	0.46 (0.30–0.72)**	**0.001**
	Yes	262 (69.9%)	113 (30.1%)	Ref.	Ref.	
BF experience	Yes	209 (70.8%)	86 (29.2%)	1.38 (0.96–1.99)	1.05 (0.70–1.59)	0.792
	No	243 (77.1%)	72 (22.9%)	Ref.	Ref.	
Breast problem	Yes	272 (82.2%)	59 (17.8%)	0.39 (0.27–0.57)	0.46 (0.28–0.75)**	**0.002**
	No	180 (64.5)	99 (35.5%)	Ref.	Ref.	
Feeding type	EBF	270 (71.6%)	107 (28.4%)	Ref.	Ref.	
	Mixed	182 (78.1%)	51 (21.9%)	0.70 (0.48–1.03)	0.83 (0.53–1.31)	0.439

Keys: *Statistically significant at *p*-values < 0.05; **at *p*-values < 0.01; ***at *p*-values < 0.001.

HI^a^, health institution; Hx^b^, history; Ref., Reference group.

## Discussion

The magnitude of effective breastfeeding among lactating women in the study area was 25.9% (95% CI: 22.47, 29.57). This result is consistent with a study conducted in Madhya Pradesh, India (31.4%) (23). However, it is lower than the findings of studies conducted in Southeast Nigeria (49%) (24), Gondar town health facilities (48%) (14), Harar city (43.4%) (25), South Ari district 36.5% (26), and Gidan district, Ethiopia (42.9%) (16). This difference could be attributed to the fact that the current study was community-based, and the district had a higher proportion of rural population than urban areas (27). Additionally, the educational levels of the participants in this study were generally lower, and a significant proportion of them were housewives. This factor might have affected the knowledge and practice of breastfeeding among the participants. Factors such as cultural beliefs, lack of awareness, and inadequate support systems could also contribute to the lower prevalence of effective breastfeeding observed in this study (28, 29).

In contrast, the magnitude of effective breastfeeding in this study is higher than that reported in a study conducted in a resettlement colony of Delhi (7.5%) (30). This difference might be attributed to several factors, including poor knowledge about the importance and benefits of effective breastfeeding, lack of breastfeeding-friendly workplaces, lack of support from family members, and harmful traditional practices. Additionally, limited access to healthcare, including comprehensive breastfeeding education and counseling programs, as well as inadequate postnatal and prenatal care for women living in resettlement areas, may contribute to this disparity (31, 32).

This study also found that working women had 1.7 times higher odds of practicing effective breastfeeding than housewives. Similar results were reported in studies conducted in Puducherry, India (33) and rural areas of Gandhinagar, India (34). This could be due to factors such as lower educational levels, reduced opportunities to attend breastfeeding-related health education sessions provided by healthcare providers, and vulnerability to traditional practices that discourage effective breastfeeding. Moreover, social isolation, lack of support from family members, lack of confidence in breastfeeding, and limited exposure to effective breastfeeding role models might result in lower effective breastfeeding practices among housewives.

Similarly, women residing in urban areas had 1.59 times higher odds of practicing effective breastfeeding techniques than those in rural areas. This could be attributed to higher educational levels, better access to breastfeeding advice, more exposure to breastfeeding role models, and access to healthcare services for any concern related to breastfeeding in urban areas.

Women aged between 26 and 30 years had 63% lower odds of practicing effective breastfeeding techniques than women aged 35 years and above. This finding is consistent with studies conducted in Southeast Nigeria (24), Puducherry, South India (33), and Gidan district, Ethiopia (16). The lower odds among younger women might be attributed to a lack of experience, lack of role models, lack of awareness about the benefit of effective breastfeeding, lack of time management due to multiple responsibilities, and their vulnerability to traditional practices.

The odds of practicing effective breastfeeding techniques were 54% lower among women who did not receive education on breastfeeding compared to those who did. This finding is also supported by studies conducted in Southeast Nigeria (24), Puducherry in South India (33), Vadodara in western India (35), and South Ari district (26), Harar city (25), Arbaminch Zuria (36), and Gondar town in Ethiopia (14). This might be because education creates awareness and promotes behavioral modifications, enabling women to understand the importance of proper breastfeeding techniques. Education also helps women anticipate challenges they may face during breastfeeding and equips them with strategies to overcome them. Moreover, it encourages women to seek support from family members, prioritize breastfeeding, and develop self-esteem, contributing to improved breastfeeding practices (18, 37, 38).

Women who initiated breastfeeding within 2 h had 73% lower odds of practicing effective breastfeeding than those who initiated breastfeeding within the first hour after giving birth. Similarly, women who breastfed after 2 h had 66% lower odds. The possible explanation for this might be that women who initiate breastfeeding later may have missed opportunities for guidance and support from healthcare professionals at the time of delivery, leading to a lack of knowledge about breastfeeding. Additional factors that can contribute to delayed initiation and lower rates of effective breastfeeding include breast problems, home deliveries, and complicated deliveries that require medical interventions. These factors can disrupt the natural process of early breastfeeding initiation and make it more challenging for women to establish effective breastfeeding practices (39–41).

Furthermore, women with current breast problems had 54% lower odds of practicing effective breastfeeding than women without breast problems. This finding is consistent with studies conducted in South Ari district (26), Harar city (25), and Gondar town (14) in Ethiopia. The possible reasons might be that women who do not practice effective breastfeeding may develop breast problems, or having breast problems may hinder their ability to practice effective breastfeeding (42).

Similar studies conducted in different parts of the world identified variables such as maternal level of education (24–26), parity (17, 26, 34, 35), antenatal care (16, 24), baby’s birth weight (34, 43), preterm birth (34, 43), early initiation of complementary feeding (36), poor knowledge on breastfeeding (36), postnatal visit (16, 25), previous breastfeeding experience (25), and place of delivery (16, 26) for having association with effective breastfeeding practice. However, these were not identified in the current study.

While interpreting the findings from this study, it is important to consider the limitations of the current study. These include the possibility that female participants may have behaved differently because they were aware of being observed despite attempts to conceal the checklists. Additionally, the outcome of effective breastfeeding practice in the neonate was not addressed in this study, such as weight gain, growth and development, malnutrition, respiratory and diarrheal diseases, and maternal–infant bonding.

## Conclusion and recommendations

Only one in four women demonstrated effective breastfeeding techniques, indicating their practice was below the WHO’s recommendation. Maternal age, women’s occupation, lack of education about breastfeeding, and experiencing breast problems were found to be significantly associated with ineffective breastfeeding practice. Therefore, providing education on breastfeeding through promoting educational programs during the antenatal and postnatal periods and early management of breast problems will benefit women in practicing effective breastfeeding techniques. It is also better for future researchers to consider interventional studies by using breastfeeding counselors.

## Data availability statement

The raw data supporting the conclusions of this article will be made available by the authors, without undue reservation.

## Ethics statement

The studies involving humans were approved by Ethical review board of Wollo University College of medicine and Health Sciences (Ref. No CMHS/004/13). The studies were conducted in accordance with the local legislation and institutional requirements. The participants provided their written informed consent to participate in this study.

## Author contributions

EM: Writing – review & editing, Writing – original draft, Visualization, Validation, Supervision, Software, Resources, Project administration, Methodology, Investigation, Formal analysis, Data curation, Conceptualization. AS: Writing – original draft, Visualization, Supervision, Resources, Project administration, Methodology, Investigation, Formal analysis. HK: Writing – review & editing, Writing – original draft, Visualization, Validation, Supervision, Software, Resources, Project administration, Methodology, Investigation, Formal analysis, Data curation, Conceptualization. MA: Writing – original draft, Visualization, Validation, Supervision, Software, Project administration, Methodology, Investigation, Data curation. AA: Writing – review & editing, Writing – original draft, Visualization, Validation, Supervision, Software, Resources, Project administration, Methodology, Investigation, Formal Analysis, Data curation, Conceptualization.
